# Development and Validation of Nomogram for Predicting Survival of Primary Liver Cancers Using Machine Learning

**DOI:** 10.3389/fonc.2022.926359

**Published:** 2022-06-20

**Authors:** Rui Chen, Beining Hou, Shaotian Qiu, Shuai Shao, Zhenjun Yu, Feng Zhou, Beichen Guo, Yuhan Li, Yingwei Zhang, Tao Han

**Affiliations:** ^1^ Department of Hepatology and Gastroenterology, Tianjin Union Medical Center, Tianjin Medical University, Tianjin, China; ^2^ Department of Hepatology and Gastroenterology, The Third Central Clinical College of Tianjin Medical University, Tianjin, China; ^3^ School of Computer Science and Technology, Dalian University of Technology, Dalian, China; ^4^ Department of Hepatology and Gastroenterology, Tianjin Union Medical Center Affiliated to Nankai University, Tianjin, China; ^5^ Department of Hepatology and Gastroenterology, Tianjin Third Central Hospital Affiliated to Nankai University, Tianjin, China; ^6^ Beijing Key Laboratory of Mobile Computing and Pervasive Device, Institute of Computing Technology, Chinese Academy of Sciences, University of Chinese Academy of Sciences, Beijing, China

**Keywords:** primary liver cancer, SEER, TCGA, nomogram, cancer specific survival

## Abstract

**Background and Aims:**

Primary liver cancer (PLC) is a common malignancy with poor survival and requires long-term follow-up. Hence, nomograms need to be established to predict overall survival (OS) and cancer-specific survival (CSS) from different databases for patients with PLC.

**Methods:**

Data of PLC patients were downloaded from Surveillance, Epidemiology, and End Results (SEER) and the Cancer Genome Atlas (TCGA) databases. The Kaplan Meier method and log-rank test were used to compare differences in OS and CSS. Independent prognostic factors for patients with PLC were determined by univariate and multivariate Cox regression analyses. Two nomograms were developed based on the result of the multivariable analysis and evaluated by calibration curves and receiver operating characteristic curves.

**Results:**

OS and CSS nomograms were based on age, race, TNM stage, primary diagnosis, and pathologic stage. The area under the curve (AUC) was 0.777, 0.769, and 0.772 for 1-, 3- and 5-year OS. The AUC was 0.739, 0.729 and 0.780 for 1-, 3- and 5-year CSS. The performance of the two new models was then evaluated using calibration curves.

**Conclusions:**

We systematically reviewed the prognosis of PLC and developed two nomograms. Both nomograms facilitate clinical application and may benefit clinical decision-making.

## Introduction

Primary liver cancer (PLC) is one of the most common malignancies of the digestive system, and its mortality rate in men and women has increased so that it now ranks fourth and seventh in terms of cancer-related deaths among global malignancies (1). Traditionally, tumors of the PLC at the pathological level can be subdivided into 3 groups: Hepatocellular carcinoma (HCC, comprising 75%-85% of cases), cholangiocarcinoma (CC, 10%-15%), and combined hepatocellular-cholangiocarcinoma (CHC) that is a rare primary liver cancer (2). Although the trend of PLC largely reflects the trend of HCC, there are notable exceptions (3). The main risk factors for liver cancer are chronic hepatitis B virus (HBV) or hepatitis C virus (HCV), eating aflatoxin-contaminated food, and heavy drinking. However, the main risk factors differ in different regions. As one of the malignant tumors, due to the low early diagnosis rate, high recurrence, and metastasis rate after resection, the 5-year survival rate of PLC has been maintained between 15% and 40% (2). With a poor prognosis and survival rates, HCC patients must have a long-term follow-up.

In the era of big data, various intelligent techniques can be used to optimize medical management plans, provide better patient care and treatment, improve population health and reduce costs (4). Surveillance, Epidemiology, and End Results (SEER) database, supported by the surveillance research program of the National Cancer Institute (NCI) Department of cancer control and Population Sciences, is one of the most representative large-scale tumor registration databases. It collects a large number of evidence-based medical data and provides systematic evidence and valuable first-hand information for clinicians’ evidence-based practice and clinical medical research (5). The Cancer Genome Atlas (TCGA) project was jointly launched by the NCI and the National Human Genome Research Institute. At present, there is clinical and genetic information of more than 11,000 tumor patients with 33 cancers of more than 20 tissue types. In addition, the fields of big data and machine learning integrate genomics and other omics, as well as electronic health records (EHRs) and other clinical data, which in turn have the potential to transform medicine. Machine learning algorithms can predict the risk of individual patients and more accurately determine which patients will benefit the most from specific treatment (6, 7).

Nomogram is a common prediction model used to predict and quantify the probability of clinical events. It is of great value for clinical decision-making and risk stratification, especially for cancer patients (8). The nomogram of breast cancer, lung cancer, liver cancer (9–11), and other malignancies can help patients to predict the risks and benefits of treatment (12) (5). In recent years, there have been relatively few systematic review studies of liver cancer by combing two separate databases. Therefore, we decided to combine SEER and TCGA databases to construct nomograms to predict the prognosis of PLC and help provide new horizons for treatment.

## Materials and Methods

### Data Collection

Clinical data were downloaded from the SEER data portal (www.seer.cancer.gov) and the TCGA data portal (https://portal.gdc.cancer.gov). Inclusion criteria included: a) complete clinical information; b) only one malignant primary tumor; c) the International Classification of Diseases for Oncology-3 (ICD-O-3) histology code: 8170/3: HCC, 8160/3: CC, 8180/3: CHC. Follow-up was suspended when patients with liver cancer died or lost contact. As SEER and TCGA data are open to the public, approval from a local ethics committee is not necessary.

The patient study variables we extracted and analyzed included baseline demographics and tumor characteristics. Baseline demographics include age(≤50y, 50–59y, 60 – 69y, 70–79y, ≥80y), race (White, Black, Other), gender (Female, Male) and time of diagnosis, survival time (months), follow-up and vital survival status. The main clinical variables were as follows: pathological type of liver cancer (HCC, CC, CHC), American Joint Committee on Cancer (AJCC) stage, and TNM staging were determined according to AJCC Cancer Staging Manual.

Overall survival (OS) or cancer-specific survival (CSS) was used as the endpoints of our study. OS represents the time duration from diagnosis to the date of death or last contact. CSS represents the time duration from diagnosis to the date of cancer death.

### Statistical Analyses

To make full use of our data to build predictive models, we used python (version 3.8) to randomize data from SEER, taking the first 9161 as the training group, and the remaining 184 as the internal validation group while 172 patients from TCGA as the external validation group. We used the training group to build the prediction model and draw the nomogram. A validation group was used to validate the model.

For survival analyses, univariate Cox analysis was used to determine significant variables, defined as a p-value of less than 0.05, from clinical data. In all statistical analyses, P values were < 0.05 is considered significant. Univariate and multivariate Cox proportional hazards regression models were used to estimate hazard ratios (HR) and corresponding 95% confidential intervals (CI) for each potential prognostic variable. SPSS 25.0 (SPSS, Chicago, IL) was used for the above analysis. Based on the results of multivariate analysis, nomograms were developed to provide visual risk prediction. The nomogram was formulated based on the results of multivariate analysis using R software. The performance of the predictive prognostic model was evaluated by calculating the concordance index (c-index). Nomograms were calibrated for one -, three -, and five-year survival rates by comparing observed survival with predicted survival probabilities.

We performed statistical analysis with R version 4.1.2 (The R Foundation for Statistical Computing, Vienna, Austria). The software packages of R Project, such as “survival(3.2-13) survminer (0.4.9)”, “survival (3.2-13 rms 6.2-0 Hmisc 4.6-0 grid 4.1.2 lattice 0.20-45 Formula 1.2-4 ggplot2 3.3.5)”, “survival (3.2-13) rms (6.2-0)” and “survival (3.2-13) timeROC (0.4)” are used to draw Kaplan–Meier (KM), nomogram and calibration diagram and timeROC, while “timeroc” and “survival” are used to verify the model and conduct AUC analysis. All packages are installed by the Packages command installed from the R language functional network CRAN.

## Results

### Patient Characteristics

According to the screening criteria, the data of 66039 patients were extracted from the SEER database. Subsequently, the data of 54588 patients were excluded because they did not have complete data. The final sample included 11451 patients in the entire cohort. Among them, 9161 (80%) patients were used as a training set to establish a predictive nomogram. The remaining 2290 (20%) patients were used to validate the nomogram. The external validation cohort included 172 patients from TCGA (**Supplementary Table 1**). The clinicopathological features of the training and validation cohort are shown in **Table 1**. All patients had complete information on survival time and cause of death. The median survival time of patients with liver cancer in this sample was 13.0 months. The 1-year, 3-year, and 5-year OS rate in the SEER population was 36.1%, 9.4%, and 2.2% respectively. While the 1 -, 3 -, and 5-year CSS were 51.5%, 29.7% and 21.5%, respectively.

**Table 1:** Characteristics of 11,451 patients with Primary Liver Cancer in SEER, n (%).

Factors	All cohort	Train cohort	Validation cohort
Total, n (%)	11451	9161	2290
**Primary diagnosis**			
HCC	10138 (88.5)	8111 (88.5)	2027 (88.5)
CC	1303 (11.4)	1043 (11.4)	260 (11.4)
CHC	10 (0.1)	7 (0.1)	3 (0.1)
**Pathologic stage**			
I	4634 (40.5)	3710 (40.5)	924 (40.3)
II	2227 (19.4)	1773 (19.4)	454 (19.8)
III	61 (0.5)	48 (0.5)	13 (0.6)
IIIA	919 (8.0)	728 (7.9)	191 (8.3)
IIIB	750 (6.5)	600 (6.5)	150 (6.6)
IIIC	190 (1.7)	150 (1.6)	40 (1.7)
IIINOS	10 (0.1)	7 (0.1)	3 (0.1)
IVA	632 (5.5)	522 (5.7)	110 (4.8)
IVB	2028 (17.7)	1623 (17.7)	405 (17.7)
**T stage**			
T0	6 (0.1)	5 (0.1)	1 (0.0)
T1	5094 (44.5)	4078 (44.5)	1016 (44.4)
T2	2212 (19.3)	1763 (19.2)	449 (19.6)
T2NOS	5 (0.0)	4 (0.0)	1 (0.0)
T2a	135 (1.2)	114 (1.2)	21 (0.9)
T2b	396 (3.5)	312 (3.4)	84 (3.7)
T3	169 (1.5)	143 (1.6)	26 (1.1)
T3NOS	14 (0.1)	11 (0.1)	3 (0.1)
T3a	1306 (11.4)	1053 (11.5)	253 (11.0)
T3b	1162 (10.1)	923 (10.1)	239 (10.4)
T4	451 (3.9)	365 (4.0)	86 (3.8)
TX	501 (4.4)	390 (4.3)	111 (4.8)
**N stage**			
N0	9905 (86.5)	7901 (86.2)	2004 (87.5)
N1	1167 (10.2)	957 (10.4)	210 (9.2)
NX	379 (3.3)	303 (3.3)	76 (3.3)
**M stage**			
M0	9423 (82.3)	7538 (82.3)	1885 (82.3)
M1	2028 (17.7)	1623 (17.7)	405 (17.7)
**Race**			
American Indian /Alaska Native	192 (1.7)	161 (1.8)	31 (1.4)
Asian or Pacific Islander	2083 (18.2)	1630 (17.8)	453 (19.8)
Black	1740 (15.2)	1415 (15.4)	325 (14.2)
White	7436 (64.9)	5955 (65.0)	1481 (64.7)
**Ethnicity**			
Non-Spanish-Hispanic-Latino	9999 (87.3)	8001 (87.3)	1998 (87.2)
Spanish-Hispanic-Latino	1452 (12.7)	1160 (12.7)	292 (12.8)
**Gender**			
Female	2910 (25.4)	2319 (25.3)	591 (25.8)
Male	8541 (74.6)	6842 (74.7)	1699 (74.2)
**Age**			
<50	692 (6.0)	541 (5.9)	151 (6.6)
50~59	3251 (28.4)	2631 (28.7)	620 (27.1)
60~69	4181 (36.5)	3349 (36.6)	832 (36.3)
70~79	2127 (18.6)	1686 (18.4)	441 (19.3)
≥80	1200 (10.5)	954 (10.4)	246 (10.7)

### Univariate and Multivariate Cox Proportional Hazard Analysis

**Figure 1:**
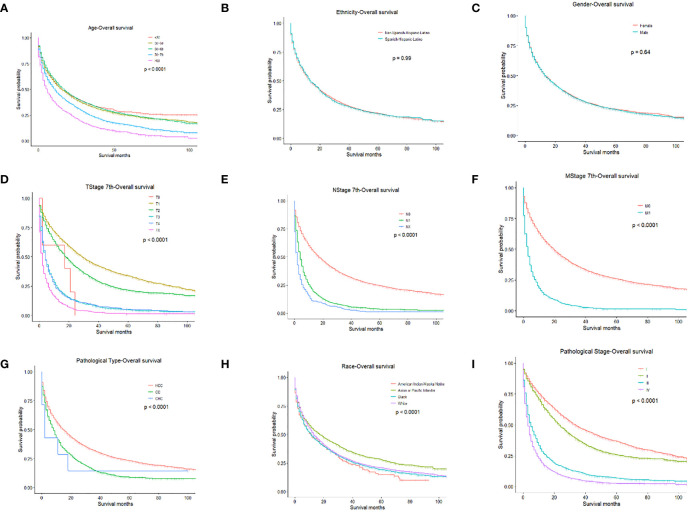
OS for PLC patients stratified by **(A)** Age, p < 0.0001; **(B)** Ethnicity p = 0.990; **(C)** Gender, p = 0.640; **(D)** T-stage, p < 0.0001; **(E)** N-stage, p < 0.0001; **(F)** M-stage, p < 0.001; **(G)** Pathological Type, p < 0.0001; **(H)** Race, p < 0.0001; **(I)** Pathological Stage, p < 0.0001.

**Figure 2:**
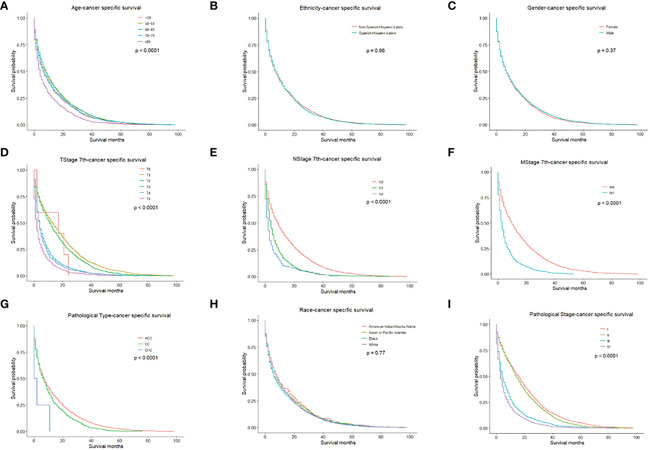
CSS for PLC patients stratified by **(A)** Age, p < 0.0001; **(B)** Ethnicity p = 0.960; **(C)** Gender, p = 0.370; **(D)** T-stage, p < 0.0001; **(E)** N-stage, p < 0.0001; **(F)** M-stage, p < 0.001; **(G)** Pathological Type, p < 0.0001; **(H)** Race, p = 0.770; **(I)** Pathological Stage, p < 0.0001.

We performed univariate and multivariate analyses to identify prognostic factors associated with the survival of PLC patients in the training cohort. In the univariate analysis, older age, higher TNM stage, higher pathologic stage, CHC, and American Indian/Alaska Native can predict worse OS and CSS. However, ethnicity and gender had no significant effect on OS or CSS (**Figures 1**, **2**).

Univariate Cox regression analysis of the training cohort revealed the role of the following parameters in predicting patient survival. Factors such as age, pathological type-CC, pathologic stage, stage T0, stage T1, and stage N were associated with patients’ prognoses. All the above variables were statistically significant (all *P*<0.05) and were included in multivariate analysis. Among these factors, the pathologic stage (c-index=0.669) and T stage (c-index=0.643) had higher discriminatory power in predicting PLC survival compared with other factors. In the Cox analysis, the maximum number of iterations was 20.

Variables involved in the multivariate analysis of OS include pathological types, pathologic stage, TNM stage, and age. According to multivariate analysis, patients with younger age, disease type of CC, lower TNM stage and adequate treatment had improved outcomes. These factors were then incorporated into the prediction model (**Table 2**).

**Table 2:** Univariate and Multivariate Cox regression for OS.

Variables	Total (%)	Univariate analysis		Multivariate analysis	
		HR (95.0%CI)	p-value	HR (95.0%CI)	p-value
	9161				
**primary_diagnosis**					
HCC	8111 (88.5)	Reference	–	Reference	–
CC	1043 (11.4)	1.520 (1.419-1.629)	0.000	0.820 (0.731-0.921)	0.001
CHC	7 (0.1)	1.582 (0.710-3.523)	0.261	1.015 (0.451-2.285)	0.972
**Pathologic stage**					
I	3710 (40.5)	Reference	–	Reference	–
II	1773 (19.4)	1.276 (1.193-1.365)	0.000	1.664 (1.389-1.992)	0.000
III	48 (0.5)	2.533 (1.873-3.426)	0.000	2.512 (1.714-3.682)	0.000
IIIA	728 (7.9)	2.626 (2.409-2.863)	0.000	1.990 (1.665-2.379)	0.000
IIIB	600 (6.5)	3.819 (3.479-4.193)	0.000	2.397 (1.999-2.875)	0.000
IIIC	150 (1.6)	3.130 (2.640-3.712)	0.000	2.561 (2.007-3.269)	0.000
IIINOS	7 (0.1)	2.468 (1.108-5.501)	0.027	1.459 (0.409-5.205)	0.560
IVA	522 (5.7)	2.900 (2.627-3.202)	0.000	2.328 (1.965-2.758)	0.000
IVB	1623 (17.7)	4.821 (4.509-5.155)	0.000	3.962 (3.496-4.489)	0.000
**T stage**					
T0	5 (0.1)	Reference	–	Reference	–
T1	4078 (44.5)	0.393 (0.163-0.945)	0.037	1.085 (0.448-2.630)	0.856
T2	1763 (19.2)	0.466 (0.193-1.121)	0.088	0.826 (0.339-2.014)	–
T2NOS	4 (0.0)	0.850 (0.228-3.165)	0.808	1.302 (0.348-4.873)	–
T2a	114 (1.2)	0.782 (0.319-1.918)	0.591	1.182 (0.478-2.921)	–
T2b	312 (3.4)	1.092 (0.451-2.643)	0.846	1.519 (0.624-3.699)	–
T3	143 (1.6)	1.063 (0.435-2.594)	0.894	1.235 (0.500-3.052)	–
T3NOS	11 (0.1)	1.095 (0.374-3.205)	0.868	1.727 (0.462-6.451)	–
T3a	1053 (11.5)	1.073 (0.445-2.583)	0.876	1.396 (0.575-3.392)	–
T3b	923 (10.1)	1.506 (0.625-3.629)	0.361	1.787 (0.735-4.342)	–
T4	365 (4.0)	1.222 (0.505-2.954)	0.657	1.334 (0.548-3.248)	–
TX	390 (4.3)	1.855 (0.768-4.483)	0.170	1.553 (0.641-3.766)	–
**N stage**					
N0	7901 (86.2)	Reference	–	Reference	–
N1	957 (10.4)	2.395 (2.230-2.571)	0.000	1.157 (1.037-1.292)	0.009
NX	303 (3.3)	3.341 (2.969-3.759)	0.000	1.150 (1.000-1.323)	0.050
**M stage**					
M0	7538 (82.3)	Reference	–		.
M1	1623 (17.7)	3.279 (3.093-3.477)			
**Race**					
American Indian /Alaska Native	161 (1.8)	Reference	–	Reference	–
Asian or Pacific Islander	1630 (17.8)	0.744 (0.623-0.889)	0.001	0.639 (0.535-0.764)	0.000
Black	1415 (15.4)	0.930 (0.778-1.111)	0.423	0.814 (0.681-0.973)	–
White	5955 (65.0)	0.877 (0.739-1.040)	0.130	0.768 (0.647-0.912)	–
**Ethnicity**					
Non-Spanish-Hispanic-Latino	8001 (87.3)	Reference	–		–
Spanish-Hispanic-Latino	1160 (12.7)	1.000 (0.933-1.073)	0.995		–
**Gender**					
Female	2319 (25.3)	Reference	–		–
Male	6842 (74.7)	1.013 (0.960-1.068)	0.644		–
**Age**					
<50	541 (5.9)	Reference	–	Reference	–
50~59	2631 (28.7)	1.078 (0.966-1.203)	0.180	1.206 (1.079-1.347)	–
60~69	3349 (36.6)	1.043 (0.936-1.162)	0.446	1.195 (1.072-1.333)	–
70~79	1686 (18.4)	1.396 (1.246-1.563)	0.000	1.557 (1.389-1.745)	0.000
≥80	954 (10.4)	1.894 (1.679-2.137)	0.000	2.265 (2.005-2.558)	0.000

### Development and Validation of a Prognostic Nomogram

**Figure 3:**
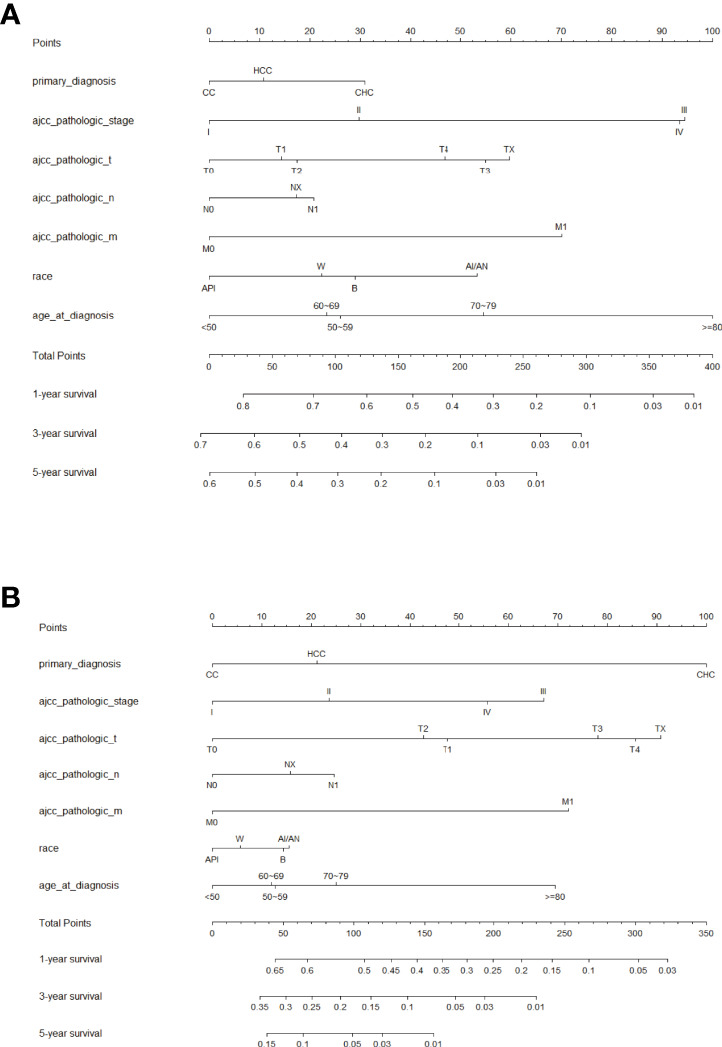
Prognostic nomogram predicting the probability of 1-, 3- and 5-year **(A)** overall survival (OS) and **(B)** cancer-specific survival (CSS). Each subtype within these significant independent variables was assigned a score on the point scale. The total score is projected to the bottom scale. API, Asian or Pacific Islander; W, White; B, Black; AI/AN, American Indian/Alaska Native.

**Figure 4:**
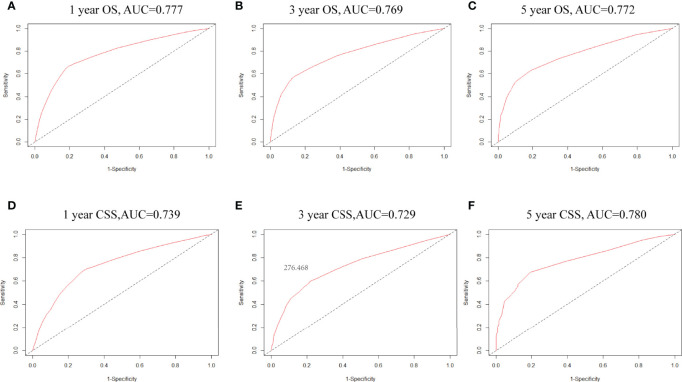
**(A–C)** ROC curves for 1-, 3- and 5-year OS based on the nomogram. The AUC was 0.777,0.834 and 0.830, respectively; **(D–F)** ROC curves for 1-,3- and 5-year CSS. The AUC was 0.739,0.729 and 0.780, respectively.

**Figure 5:**
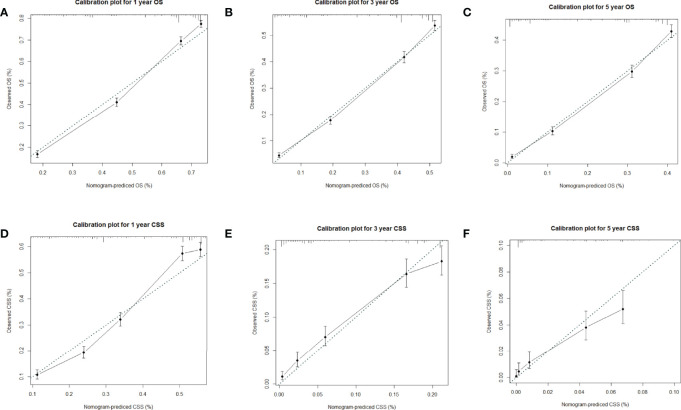
**(A–C)** Calibration plots for 1-,3- and 5-year OS in the training cohort; **(D**–**F)** Calibration plots for 1-,3- and 5-year CSS in the training cohort.

Factors from the multivariate analysis were used to develop nomograms to calculate 1-,3 -, and 5-year OS or CSS probabilities (**Figure 3**). Each prognostic parameter was scored according to its prognostic value. The total score was used to predict 1 -, 3 -, and 5-year OS and CSS. Furthermore, the total score for all variables was converted into an estimate of the probability of death. The distinction between survival probabilities and actual observations was assessed using the c-index. The value of the c-index fluctuates between 0.5 and 1.0 representing random chance and 1.0 represents fully corrected discrimination (13). The c-index of the prognostic nomogram for OS prediction was 0.702 (95% CI, 0.696–0.708) in the training cohort and 0.702 (95% CI, 0.689–0.714) in the internal validation cohort. We tested the nomogram using an internal receiver operating characteristic (ROC) curve in the training cohort. The area under the curve (AUC) was 0.777, 0.769 and 0.772 for 1-,3- and 5-year OS respectively, with 0.739, 0.729 and 0.780 for 1-,3- and 5-year CSS (**Figure 4**). The calibration plot shows good agreement between the internal and external validation cohorts (**Figure 5**) (**Supplementary Figures 1**, **2**).

## Discussion

Worldwide, PLC is a common cause of cancer-related death. PLC death rates are increasing faster than any other cancer (14). In addition, PLC is the second most lethal tumor after pancreatic cancer. HCC accounts for the majority of PLC (15). The increasing number of deaths due to HCC is an increasing concern (16). Disease and tumor-related factors have a great impact on the treatment of PLC (17). CC is an epithelial cell malignancy, and most CCs are well, moderately, and poorly differentiated adenocarcinomas, with other histological subtypes, rarely occurring. Most CCs are new-onset, with no risk factors identified (18). Moreover, CHC is a rare and aggressive variant with features of both HCC and CC, and it is unclear whether treatments commonly used for PLC are effective. The prognosis of CHC is particularly poor due to its aggressive nature. The estimated incidence of CHC ranges from 1% to 14.2% (19) (20).

In this research, patients diagnosed with PLC were included in the analysis. With more than 60000 patients, we included 9161 patients with complete clinical information in the training set from the SEER database and 172 patients from TCGA. By univariate analysis, race, age, pathologic stage, primary diagnosis, and T and N stage were all related to liver cancer progression. In addition, we conducted the multivariate analysis using these significant variables in univariate analysis. In multivariable analyses, we demonstrated that older age, higher pathological stage, and more advanced T and N stages were independently associated with poor overall survival in PLC.

PLC incidence rates vary by race/ethnicity and state, largely because of differences in the prevalence of major risk factors and, to some extent, because of different access to high-quality care (21) (22). We can also know that social status is associated with better survival. In this research, we analyzed the association between ethnicity and race with tumor survival and found that survival was slightly lower in the American Indian/Alaska Native and black.

Many studies have shown that the TNM stage may be an important prognostic factor in HCC (23) (24). In the present study, we analyzed the relationship between the TNM stage and tumor survival and found that the higher the TNM stage, the worse the survival.

Hence, we plotted the nomogram according to independent prognostic factors in the multivariate. The data used were derived from the SEER database, which ensured the validity and reliability of our conclusions, as well as the internal and external validity of the nomograms. To validate this value and prevent overfitting of the current model, it is necessary to validate a new nomogram. Moreover, we validated the predictive value of the model by using both internal and external validation cohorts. In addition, we measured the accuracy of this model by ROC curve and a calibration plot, and the larger the AUC, the higher the accuracy of the model. The training cohort AUC was 0.777,0.769 and 0.772 for 1-,3- and 5-year OS and 0.739,0.729 and 0.780 for 1-,3- and 5-year CSS. All these results indicated that the model had good accuracy for the prediction of liver cancer survival. Meanwhile, the calibration curve also validated the model’s prediction ability on the overall sample.

It has been reported that individualized prediction is considered a critical condition of predictive models (25). However, most current studies are based on a single database (26) (27). In this research, we mainly performed long-term follow-ups of patients with PLC. The main objective of this study was to use two databases to predict total and cancer-specific mortality in patients with liver cancer, which differs from currently published studies regarding predictive nomograms. The huge number of patients with PLC recorded in the SEER database helped us to build a more accurate model. In addition, the items included in the nomogram are common, easily accessible, and comprehensible items for physicians and patients in the clinic.

There are also relevant studies applied to predict cancer-specific diseases. Ni et al. (5) developed a hepatocellular carcinoma nomogram to predict cancer-specific mortality and overall mortality using the SEER database, which will help clinicians to obtain personal prediction information to determine whether patients are at high risk of death. Song et al. (28) created a pancreatic cancer survival nomogram to effectively predict patients’ survival and use it in clinical practice. Similarly, Wang et al. (29) developed and validated a new nomogram for pulmonary invasive mucinous adenocarcinoma based on the SEER database, which is expected to provide new ideas for treatment. All of these studies are based on a bioinformatics database such as the SEER database to develop nomograms for multiple cancers that predict CSS characteristics to help clinicians make clinical decisions. In this research, we used two bioinformatics databases (SEER and TCGA databases) and developed two nomograms simultaneously. Making clinical decisions more convenient and effective.

Although we have developed powerful nomograms, there are still several limitations that must be acknowledged. Potential prognostic factors available in public databases are limited. Further analysis with a more complete data set may enhance the predictive power of this tool. Data from SEER and TCGA that did not report underlying chronic liver disease, laboratory studies to assess liver function, calculation of Child-Pugh score, or details of tumor characteristics were missing, which would be important for further treatment and thus impact survival. However, data from the multicenter nature of the sources provide significant benefits. This model comprehensively evaluated the clinical features and treatment of liver cancer and provided ideas for improving the prognosis of liver cancer.

In conclusion, we conducted an analysis of the prognosis of PLC based on a large population in the SEER and TCGA databases. Reviewed the prognosis of PLC and developed and validated two new nomograms. We then elucidated the factors influencing the prognosis of PLC. These models give us a deeper understanding of PLC. They are expected to be used as stratification tools in clinical studies and as evidence for the development of interventions to improve survival.

## Data Availability Statement

The original contributions presented in the study are included in the article/**Supplementary Material**. Further inquiries can be directed to the corresponding authors.

## Ethics Statement

This study was based on publicly available data from the SEER and TCGA database, and did not involve interaction with human subjects or the use of personally identifiable information. The study did not require informed consent for SEER and TCGA registration cases, and the author obtained a”limited use data agreement” from SEER. No trial registration was required.

## Author Contributions

Software, formal analysis, investigation, and writing of the original draft (YZ and TH), acquisition of the data (BH), analysis and interpretation of data (SS, ZY, and SQ), drafted the manuscript (RC), critical revision of the manuscript for important intellectual content (BG and FZ), and study supervision (YL). All authors reviewed and commented on the manuscript and approved the final version.

## Conflict of Interest

The authors declare that the research was conducted in the absence of any commercial or financial relationships that could be construed as a potential conflict of interest.

## Publisher’s Note

All claims expressed in this article are solely those of the authors and do not necessarily represent those of their affiliated organizations, or those of the publisher, the editors and the reviewers. Any product that may be evaluated in this article, or claim that may be made by its manufacturer, is not guaranteed or endorsed by the publisher.

## Supplementary Material

Supplementary Figure 1 **(A–C)** Calibration plots for 1-,3- and 5-year OS in the internal validation cohort; **(D–F)** Calibration plots for 1-,3- and 5-year CSS in the internal validation cohort.

Supplementary Figure 2 **(A–C)** Calibration plots for 1-,3- and 5-year OS in the external validation cohort.

Supplementary Table 1 Characteristics of 172 patients with Primary Liver Cancer in TCGA.

## Abbreviations

PLC, Primary liver cancer; HCC, Hepatocellular carcinoma; CC, Cholangiocarcinoma; CHC, Combined Hepatocellular-Cholangiocarcinoma; HBV, Chronic hepatitis B virus; HCV, Hepatitis C virus; SEER, Surveillance, Epidemiology, and End Results; NCI, National Cancer Institute; EHRs, The Cancer Genome Atlas (TCGA); electronic health records; OS, Overall survival; CSS, Cancer-specific survival.
