# Supplementary figures and images for: Development and Validation of Nomogram for Predicting Survival of Primary Liver Cancers Using Machine Learning
Source: Front Oncol. 2022 Jun 20;12:926359. doi: 10.3389/fonc.2022.926359 (PMC9258303; doi:10.3389/fonc.2022.926359)

A

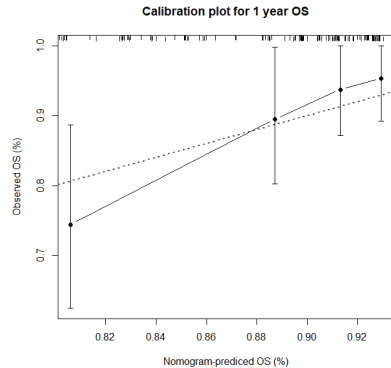

B

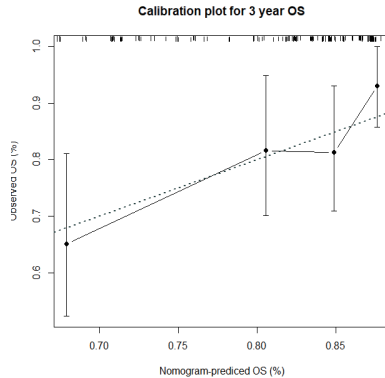

C

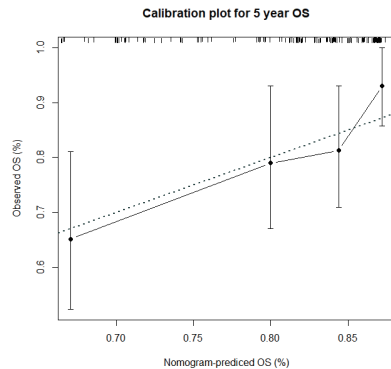

Supplement: Supplementary Figure 1 — (A–C) Calibration plots for 1-,3- and 5-year OS in the internal validation cohort; (D–F) Calibration plots for 1-,3- and 5-year CSS in the internal validation cohort. [file DataSheet_1.pdf]

A

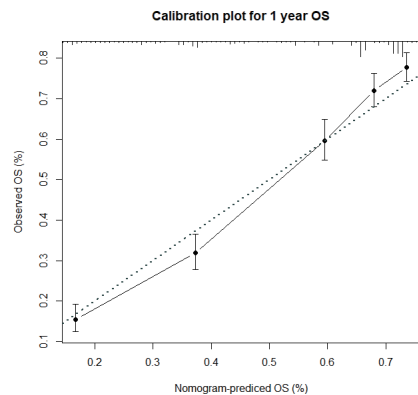

B

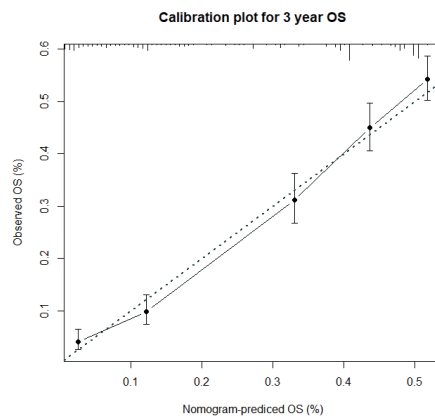

C

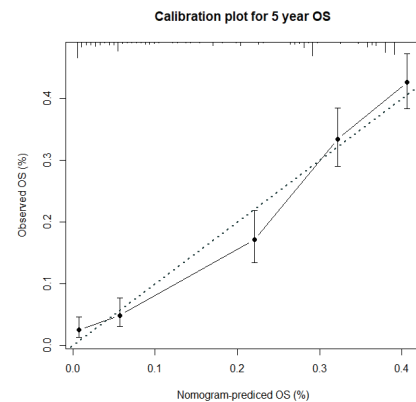

D

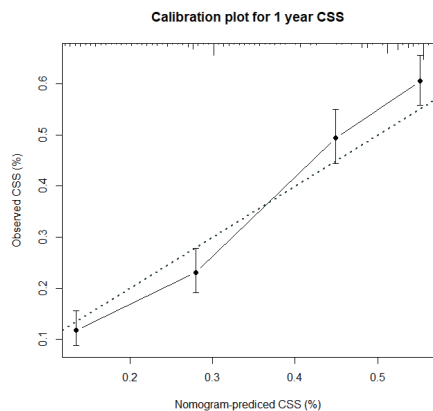

E

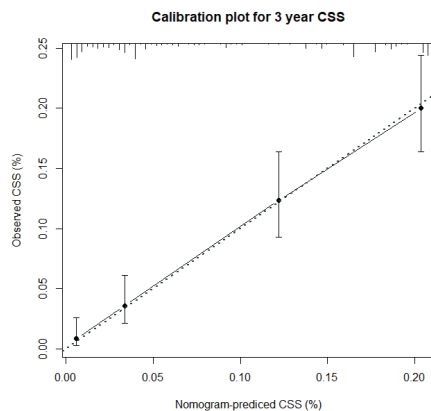

F

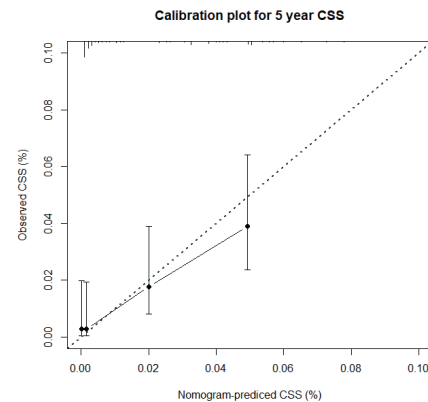

Supplement: Supplementary Figure 2 — (A–C) Calibration plots for 1-,3- and 5-year OS in the external validation cohort. [file DataSheet_2.pdf]
