# Supplementary material for: Development and Validation of Nomogram for Predicting Survival of Primary Liver Cancers Using Machine Learning
Source: Front Oncol. 2022 Jun 20;12:926359. doi: 10.3389/fonc.2022.926359 (PMC9258303; doi:10.3389/fonc.2022.926359)
Supplement: Supplementary Table 1 — Characteristics of 172 patients with Primary Liver Cancer in TCGA. [file Table_1.docx]

| **Factors** | **All cohort** |
| --- | --- |
| Total, n (%) | 172 |
| primary_diagnosis |  |
| HCC | 138 (80.2) |
| CC | 30 (17.4) |
| CHC | 4 (2.3) |
| Ajcc_pathologic_stage |  |
| Ⅰ | 85 (49.4) |
| Ⅱ | 46 (26.7) |
| Ⅲ | 2 (1.2) |
| ⅢA | 23 (13.4) |
| ⅢB | 4 (2.3) |
| ⅢC | 6 (3.5) |
| ⅣA | 3 (1.7) |
| ⅣB | 3 (1.7) |
| Ajcc pathologic_T |  |
| T1 | 85 (49.4) |
| T2 | 42 (24.4) |
| T2a | 3 (1.7) |
| T2b | 3 (1.7) |
| T3 | 10 (5.8) |
| T3a | 19 (11.0) |
| T3b | 3 (1.7) |
| T4 | 7 (4.1) |
| Ajcc pathologic_N |  |
| N0 | 144 (83.7) |
| N1 | 5 (2.9) |
| NX | 23 (13.4) |
| Ajcc_pathologic_M |  |
| M0 | 169 (98.3) |
| M1 | 3 (1.7) |
| race |  |
| American Indian/Alaska Native | 1 (0.6) |
| Asian or Pacific Islander | 90 (52.3) |
| Black | 5 (2.9) |
| White | 76 (44.2) |
| ethnicity |  |
| Non-Spanish-Hispanic-Latino | 167 (97.1) |
| Spanish-Hispanic-Latino | 5 (2.9) |
| Gender |  |
| Female | 67 (39.0) |
| Male | 105 (61.0) |
| Age_at_diagnosis |  |
| <50 | 27 (15.7) |
| 50~59 | 47 (27.3) |
| 60~69 | 56 (32.6) |
| 70~79 | 36 (20.9) |
| ≥80 | 6 (3.5) |
