# Supplementary material for: Development and Validation of Nomogram for Predicting Survival of Primary Liver Cancers Using Machine Learning
Source: Front Oncol. 2022 Jun 20;12:926359. doi: 10.3389/fonc.2022.926359 (PMC9258303; doi:10.3389/fonc.2022.926359)
Supplement: Supplementary file 4 [file Table_2.docx]

Table 2 Univariate and Multivariate Cox regression for OS

| **Variables** | **Total(%)** | **Univariate analysis** | | **Multivariate analysis** | |
| --- | --- | --- | --- | --- | --- |
|  |  | **HR(95.0%CI)** | **p value** | **HR(95.0%CI)** | **p value** |
|  | 9161 |  |  |  |  |
| **primary_diagnosis** |  |  |  |  |  |
| HCC | 8111 (88.5) | Reference | - | Reference | - |
| CC | 1043 (11.4) | 1.520(1.419-1.629) | 0.000 | 0.820(0.731-0.921) | 0.001 |
| CHC | 7 (0.1) | 1.582(0.710-3.523) | 0.261 | 1.015(0.451-2.285) | 0.972 |
| **Pathologic stage** |  |  |  |  |  |
| Ⅰ | 3710 (40.5) | Reference | - | Reference | - |
| Ⅱ | 1773 (19.4) | 1.276(1.193-1.365) | 0.000 | 1.664(1.389-1.992) | 0.000 |
| Ⅲ | 48 (0.5) | 2.533(1.873-3.426) | 0.000 | 2.512(1.714-3.682) | 0.000 |
| ⅢA | 728 (7.9) | 2.626(2.409-2.863) | 0.000 | 1.990(1.665-2.379) | 0.000 |
| ⅢB | 600 (6.5) | 3.819(3.479-4.193) | 0.000 | 2.397(1.999-2.875) | 0.000 |
| ⅢC | 150 (1.6) | 3.130(2.640-3.712) | 0.000 | 2.561(2.007-3.269) | 0.000 |
| ⅢNOS | 7 (0.1) | 2.468(1.108-5.501) | 0.027 | 1.459(0.409-5.205) | 0.560 |
| ⅣA | 522 (5.7) | 2.900(2.627-3.202) | 0.000 | 2.328(1.965-2.758) | 0.000 |
| ⅣB | 1623 (17.7) | 4.821(4.509-5.155) | 0.000 | 3.962(3.496-4.489) | 0.000 |
| **T stage** |  |  |  |  |  |
| T0 | 5 (0.1) | Reference | - | Reference | - |
| T1 | 4078 (44.5) | 0.393(0.163-0.945) | 0.037 | 1.085(0.448-2.630) | 0.856 |
| T2 | 1763 (19.2) | 0.466(0.193-1.121) | 0.088 | 0.826(0.339-2.014) | - |
| T2NOS | 4 (0.0) | 0.850(0.228-3.165) | 0.808 | 1.302(0.348-4.873) | - |
| T2a | 114 (1.2) | 0.782(0.319-1.918) | 0.591 | 1.182(0.478-2.921) | - |
| T2b | 312 (3.4) | 1.092(0.451-2.643) | 0.846 | 1.519(0.624-3.699) | - |
| T3 | 143 (1.6) | 1.063(0.435-2.594) | 0.894 | 1.235(0.500-3.052) | - |
| T3NOS | 11 (0.1) | 1.095(0.374-3.205) | 0.868 | 1.727(0.462-6.451) | - |
| T3a | 1053 (11.5) | 1.073(0.445-2.583) | 0.876 | 1.396(0.575-3.392) | - |
| T3b | 923 (10.1) | 1.506(0.625-3.629) | 0.361 | 1.787(0.735-4.342) | - |
| T4 | 365 (4.0) | 1.222(0.505-2.954) | 0.657 | 1.334(0.548-3.248) | - |
| TX | 390 (4.3) | 1.855(0.768-4.483) | 0.170 | 1.553(0.641-3.766) | - |
| **N stage** |  |  |  |  |  |
| N0 | 7901 (86.2) | Reference | - | Reference | - |
| N1 | 957 (10.4) | 2.395(2.230-2.571) | 0.000 | 1.157(1.037-1.292) | 0.009 |
| NX | 303 (3.3) | 3.341(2.969-3.759) | 0.000 | 1.150(1.000-1.323) | 0.050 |
| **M stage** |  |  |  |  |  |
| M0 | 7538 (82.3) | Reference | - |  | . |
| M1 | 1623 (17.7) | 3.279(3.093-3.477) |  |  |  |
| **Race** |  |  |  |  |  |
| American Indian  /Alaska Native | 161 (1.8) | Reference | - | Reference | - |
| Asian or Pacific Islander | 1630 (17.8) | 0.744(0.623-0.889) | 0.001 | 0.639(0.535-0.764) | 0.000 |
| Black | 1415 (15.4) | 0.930(0.778-1.111) | 0.423 | 0.814(0.681-0.973) | - |
| White | 5955 (65.0) | 0.877(0.739-1.040) | 0.130 | 0.768(0.647-0.912) | - |
| **Ethnicity** |  |  |  |  |  |
| Non-Spanish-Hispanic-Latino | 8001 (87.3) | Reference | - |  | - |
| Spanish-Hispanic-Latino | 1160 (12.7) | 1.000(0.933-1.073) | 0.995 |  | - |
| **Gender** |  |  |  |  |  |
| Female | 2319 (25.3) | Reference | - |  | - |
| Male | 6842 (74.7) | 1.013(0.960-1.068) | 0.644 |  | - |
| **Age** |  |  |  |  |  |
| <50 | 541 (5.9) | Reference | - | Reference | - |
| 50~59 | 2631 (28.7) | 1.078(0.966-1.203) | 0.180 | 1.206(1.079-1.347) | - |
| 60~69 | 3349 (36.6) | 1.043(0.936-1.162) | 0.446 | 1.195(1.072-1.333) | - |
| 70~79 | 1686 (18.4) | 1.396(1.246-1.563) | 0.000 | 1.557(1.389-1.745) | 0.000 |
| ≥80 | 954 (10.4) | 1.894(1.679-2.137) | 0.000 | 2.265(2.005-2.558) | 0.000 |
